# A new analytical model for flow in acidized fractured-vuggy porous media

**DOI:** 10.1038/s41598-019-44802-2

**Published:** 2019-06-05

**Authors:** Gang Lei, Qinzhuo Liao, Dongxiao Zhang

**Affiliations:** 1 Department of Petroleum Engineering, CPG, King Fahd University of Petroleum and Minerals, Dhahran, Saudi Arabia; 2 ERE & BIC-ESAT, College of Engineering, Peking University, Beijing, China

**Keywords:** Hydrology, Limnology

## Abstract

Acidizing is one of the widely used technologies that makes the development of naturally fractured-vuggy reservoirs effective. During the process of acidizing, carbonate minerals are dissolved by hydrochloric acid, which can create high conductivity channels and wormholes to connect fractures and pores. In this work, a new analytical model, incorporating the heterogeneity of the pore networks into acidizing region, is proposed to study the flow characteristics in acidized fractured-vuggy reservoirs. The model is coupled by an acidized inner region and a conceptualized outer region of common triple medium. The porosity and permeability of inner region, which are rather heterogeneous and disordered when observed at different length scales, can be well addressed by fractal theory. The properties of the outer region can be described with three basic parameters: the matrix block size *L*_M_, the space interval of fracture *L*_F_ and the radius of the vug *L*_v_. Results show that the flow characteristic curves can be characterized by six flow stages (i.e. wellbore storage stage, radial flow stage in the interior region, fracture-vug inter-porosity flow stage, transition flow stage, fracture-matrix inter-porosity flow stage and external boundary response stage). It can be applied to estimate reservoir parameters for uncertainty reduction using inverse modeling.

## Introduction

In the past few decades, characterizing the fluid flow mechanism in fractured-vuggy porous media has become a hot topic in petroleum engineering, as increasing carbonate reservoirs are discovered worldwide^1–6^. It is reported that fractured-vuggy reservoirs have complex pore systems including matrix, fractures and vugs which cause the fluid flow characteristics within these reservoirs more complicated than that in conventional sandstones^7–11^.

At present, acidizing or acid fracturing stimulation technology is essential for the development of fractured-vuggy carbonate reservoirs^12,13^. As acid is injected, carbonate minerals are dissolved and highly conductive channels or wormholes are usually formed^14^. Wormhole forming mechanism in carbonate porous media has been widely investigated using experimental and theoretical methods^15–21^. Many scholars have suggested acid flow in the pore networks selectively and create different types of wormhole structures^17,20^. Mostofizadeh and Economides^15^ carried out radial acidizing tests on limestone rocks and suggested there exist an optimum injection rate. Frick *et al*.^16^ conducted radial core experiments to investigate the interaction between hydrochloric acid and limestones and identified the optimum injection rate. Fredd^17^ presented a dynamic mathematical model for wormhole formation determine, and suggested the derived model can be used to determining optimal acidizing strategies. Tremblay^18^ proposed a sand transport model to calculate the wormhole growth. Liu *et al*.^19^ derived a coupled model to study the wormhole propagation behavior under reservoir condition and they concluded that, due to the effect of the compressed zone, there existed a maximum wormhole length for both constant rate condition and constant pressure condition. Zakaria^20^ conducted various laboratory tests to study the acid stimulation treatments in carbonate rocks and suggested that acid stimulation treatments are significantly affected by the pore heterogeneity and the acid propagate through the fractured-vuggy carbonate rocks in a selective manner. Li *et al*.^21^ studied the moving interface of acid fingering using both the experimental tests and numerical method. Dejam *et al*.^22–27^ formulated mathematical models to study the solute transport and dispersion in porous media with coupled systems. In addition, a theoretical model was derived by Ugursal^28^ to evaluate acid fracturing performances in carbonate rocks.

Recently, the fluid flow and transport behavior in fractured-vuggy porous media have been analyzed with theoretical methods^3,9,29–34^. Wu *et al*.^29–31^ investigated multiphase flow in fractured-vuggy reservoirs using triple-continuum models. Yao *et al*.^32^ presented discrete fracture-vug network model (DFVN) modeling fluid flow in carbonate rocks. However, to the best of my knowledge, reliable characterization of actual pore networks in acidized region is severely limited, and the corresponding analytical models for acidizing wells with wormholes are scarce. In 2014, Wang *et al*.^33^ derived a theoretical model considering acid wormholes to recognize the flow characteristics. And in their model, the pore networks in acidized region were conceptualized as multi-branched wormholes (i.e., the pore networks in acidized region were simplified as multi-branched high-permeability channels), which were used to bridge the gap between the outer region of triple medium and the wellbore. In 2018, Wang *et al*.^34^ also investigated flow characteristics in fractured-vuggy carbonate reservoirs using semi-analytical model considering wormholes as multi-branched fractures. Other scholars proposed composite model to study the pressure transient characteristics in fractured-vuggy reservoirs within which the pore networks in acidized region are equivalent to homogeneous medium of Euclidean geometry^35,36^. Generally speaking, due to the interaction and connectivity between the wormholes and the matrix-fracture-vug systems, the acidized region with multiple property scales and non-Euclidean pore network^37^ was highlighted by the characteristics of heterogeneity, which increases the research complexity. As a result, developing an applicable analytical model solving this challenge is more desirable.

**Figure 1:**
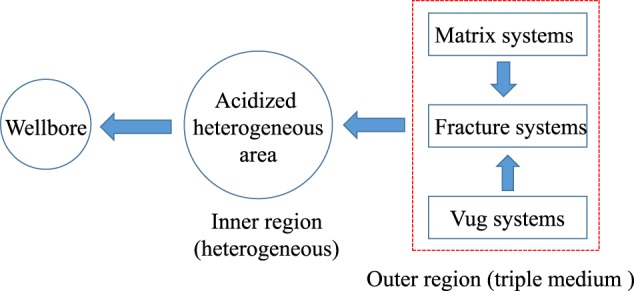
Schematic diagram of the physical model.

It is reported that reservoir heterogeneity provides uncertainty in fluid flow through porous media, so, in order to provide a more realistic representation of flow in acidized fractured-vuggy reservoirs, the heterogeneity of the pore networks in acidized region should be taking into consideration. However, previous models neglect the heterogeneity in acidized region. In this paper, we derive an analytical solution by considering the heterogeneity of the pore networks in acidized region to analyze flow characteristic in fractured-vuggy carbonate reservoirs. In this presented model, pore networks properties (porosity and permeability) in acidized region recognized as an inner zone is modeled based on the fractal geometry theory. The schematic diagram of the physical model is shown in Fig. 1. Compared with the previous models, our model takes the heterogeneity of the pore networks in acidized region into account. The derived model can help to reduce the uncertainty in flow through porous media and obtain data with high accuracy.

As the outline of this work, the theoretical models and the corresponding analytical solution in Laplace space are provided in the following section. Then, the results will be analyzed, followed by the discussions of the derived model. Finally, the conclusions are presented.

## Methods

### Physical concept

**Figure 2:**
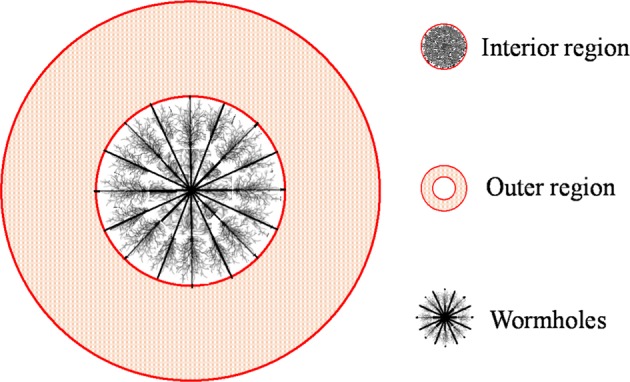
Schematic of the fractured-vuggy physical model.

As observed in the carbonate formation of the Tahe and Tarim Oilfield in western China, fractured-vuggy reservoirs typically consist of matrix, fracture and vug systems^5^. It is reported that acidizing or acid fracturing stimulation technology has become an important stimulation measure, which is essential to improve or recover productivity for fractured-vuggy carbonate reservoirs^13,38,39^. During the process of acidizing, wormhole dissolution patterns will be affected by a series of complicated physical chemical transportations and reactions^40,41^. As shown in Fig. 2 (i.e. the schematic of the fractured-vuggy model), the physical model was conceptualized as a composite model, which could be divided into the acidized region (interior region) and outer region. In the acidized region, the complicated wormholes are usually connected with matrix-vug-fracture system which is treated as a fractal medium. And the outer region is treated as a common triple medium^29,31,42^. Using the conceptual model, the new composite model is derived under the following assumptions:

1. As fractal geometry theory is a natural candidate for the representation of heterogeneity of the acidized region^43–45^, fractal geometry theory is applied in this article to account for the pore network in the acidized region. However, the outer region is assumed as a common triple medium, in which matrix and vug are regarded as the main sources of hydrocarbon, and fracture is treated as the flow path^35,46^. The formation effective thickness is constant.
2. During the flow, the property parameters, such as permeability, porosity and fluid viscosity, are constant. Gravity is neglected. Moreover, the isothermal and Darcy flow process is assumed.

### Mathematical model

**Figure 3:**
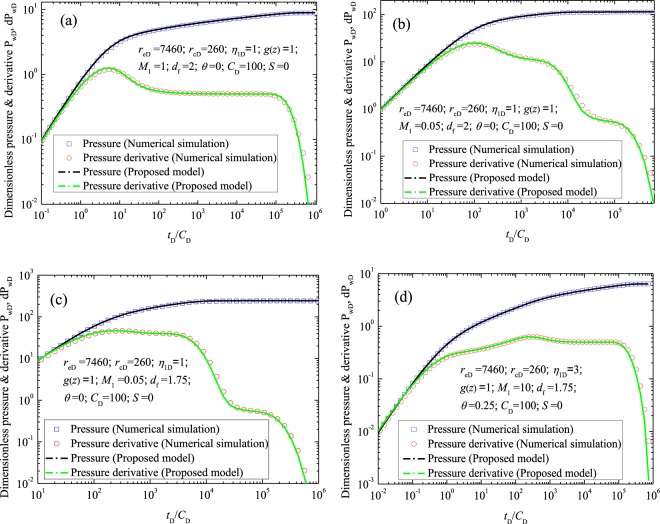
Comparison of *P*_wD_ and d*P*_wD_ obtained from the proposed model and numerical simulation: (**a**) case 1; (**b**) case 2; (**c**) case 3; (**d**) case 4.

As depicted in Fig. 3, the composite model can be divided into the acidized region (interior region) and outer region. Based on fractal geometry theory, the permeability and porosity in the interior region can be expressed as^47–49^

1 $$k={k}_{1}{(\frac{r}{{r}_{{\rm{w}}}})}^{{d}_{{\rm{f}}}-\theta -2};\,\phi ={\phi }_{1}{(\frac{r}{{r}_{{\rm{w}}}})}^{{d}_{{\rm{f}}}-2}$$

where *k*_1_ is the permeability at the edge of the wellbore, *φ*_1_ is porosity at the edge of the wellbore, *r*_w_ is wellbore radius, *d*_f_ is pore spaces fractal dimension, *θ* is the conductivity index^49,50^.

Based on Eq. (1), the corresponding governing equation in interior region is

2 $$\begin{array}{ll}\frac{{\partial }^{2}{p}_{1}}{\partial {r}^{2}}+\frac{{d}_{{\rm{f}}}-\theta -1}{r}\frac{\partial {p}_{1}}{\partial r}=\frac{{C}_{{\rm{t}}}\mu {\phi }_{1}}{{k}_{1}}{(\frac{r}{{r}_{{\rm{w}}}})}^{\theta }\frac{\partial {p}_{1}}{\partial t} & ({r}_{{\rm{w}}}\le r\le {r}_{{\rm{c}}})\end{array}$$

in which *p*_1_ is the pressure in interior region, *μ* is the fluid viscosity, *C*_t_ is the rock compressibility in interior region, *r*_c_ is the outer boundary of the acidized region, and *t* is the time.

In outer region, the corresponding governing equation can be given by

3 $$\{\begin{array}{ll}\frac{{k}_{{\rm{F}}}}{\mu }\frac{1}{r}\frac{\partial }{\partial r}(r\frac{\partial {p}_{{\rm{F}}}}{\partial r})-{C}_{{\rm{M}}}{\phi }_{{\rm{M}}}\frac{\partial {p}_{{\rm{M}}}}{\partial t}-{C}_{{\rm{v}}}{\phi }_{{\rm{v}}}\frac{\partial {p}_{{\rm{v}}}}{\partial t}={C}_{{\rm{F}}}{\phi }_{{\rm{F}}}\frac{\partial {p}_{{\rm{F}}}}{\partial t} & ({r}_{{\rm{c}}}\le r\le {r}_{{\rm{e}}})\\ {C}_{{\rm{M}}}{\phi }_{{\rm{M}}}\frac{\partial {p}_{{\rm{M}}}}{\partial t}=\frac{{\alpha }_{{\rm{FM}}}{k}_{{\rm{M}}}}{\mu }({p}_{{\rm{F}}}-{p}_{{\rm{M}}}) & ({r}_{{\rm{c}}}\le r\le {r}_{{\rm{e}}})\\ {C}_{{\rm{v}}}{\phi }_{{\rm{v}}}\frac{\partial {p}_{{\rm{v}}}}{\partial t}=\frac{{\alpha }_{{\rm{Fv}}}{k}_{{\rm{v}}}}{\mu }({p}_{{\rm{F}}}-{p}_{{\rm{v}}}) & ({r}_{{\rm{c}}}\le r\le {r}_{{\rm{e}}})\end{array}$$

where *r*_e_ is the outer boundary of the reservoir, the subscript F, M and v represent the fracture system, matrix system and vug system. *k*_τ_ (τ = F, M and v) is the permeability in outer region. *p*_τ_ (τ = F, M and v) is the pressure in outer region. *φ*_M_ is matrix porosity, *φ*_F_ is fracture porosity and *φ*_v_ is vug porosity. *C*_M_ is matrix compressibility, *C*_F_ is fracture compressibility and *C*_v_ is vug compressibility. *α*_FM_ and *α*_Fv_ are shape factors for fracture-vug and fracture-matrix, respectively. More details about the calculation equations for physical parameters in triple medium, such as *k*_F_, *k*_v_, *φ*_F_, *φ*_v_, *α*_FM_ and *α*_Fv_, can be found in Appendix A.

At the initial state, the following pressure equation can be written as

4 $${p}_{{\rm{F}}}(r,t=0)={p}_{{\rm{M}}}(r,t=0)={p}_{{\rm{v}}}(r,t=0)={p}_{0}$$

where *p*_0_ is the initial pressure.

Three types of external boundary conditions (constant pressure boundary, infinite boundary or closed boundary) can be expressed respectively as^5^

5a $${p}_{{\rm{F}}}(r={r}_{{\rm{e}}},t)={p}_{0}$$

5b $${p}_{{\rm{F}}}(r={r}_{{\rm{e}}}\to \infty ,t)={p}_{0}$$

5c $${\frac{\partial {p}_{{\rm{F}}}}{\partial r}|}_{r={r}_{{\rm{e}}}}=0$$

The inner boundary condition can be expressed as

6 $${\frac{2{\rm{\pi }}{k}_{1}h}{\mu }(r\frac{\partial {p}_{{\rm{1}}}}{\partial r})|}_{r={r}_{{\rm{w}}}}=qB$$

where *h* is effective thickness, *B* is fluid volume factor, and *q* is the flow rate at wellhead, which is positive for producers and negative for injectors.

The interface continuity conditions are governed by

7 $$\{\begin{array}{l}{{p}_{{\rm{1}}}|}_{r={r}_{{\rm{c}}}}={{p}_{{\rm{F}}}|}_{r={r}_{{\rm{c}}}}\\ {\frac{{k}_{{\rm{1}}}}{\mu }{(\frac{{r}_{{\rm{c}}}}{{r}_{{\rm{w}}}})}^{{d}_{{\rm{f}}}-\theta -2}\frac{\partial {p}_{{\rm{1}}}}{\partial r}|}_{r={r}_{{\rm{c}}}}={\frac{{k}_{{\rm{F}}}}{\mu }\frac{\partial {p}_{{\rm{F}}}}{\partial r}|}_{r={r}_{{\rm{c}}}}\end{array}$$

By using dimensionless variables listed in Table 1, Eqs (2–7) can be rewritten as following:

8 $$\{\begin{array}{c}\frac{{\partial }^{2}{p}_{{\rm{1D}}}}{\partial {r}_{{\rm{D}}}^{2}}+\frac{{d}_{{\rm{f}}}-\theta -1}{{r}_{{\rm{D}}}}\frac{\partial {p}_{{\rm{1D}}}}{\partial {r}_{{\rm{D}}}}=\frac{{\eta }_{{\rm{1D}}}}{{M}_{1}}{r}_{{\rm{D}}}^{\theta }\frac{\partial {p}_{{\rm{1D}}}}{\partial {t}_{{\rm{D}}}}\\ \frac{1}{{r}_{{\rm{D}}}}\frac{\partial }{\partial {r}_{{\rm{D}}}}({r}_{{\rm{D}}}\frac{\partial {p}_{{\rm{FD}}}}{\partial {r}_{{\rm{D}}}})-{\lambda }_{{\rm{FM}}}({p}_{{\rm{FD}}}-{p}_{{\rm{MD}}})-{\lambda }_{{\rm{Fv}}}({p}_{{\rm{FD}}}-{p}_{{\rm{vD}}})={\eta }_{{\rm{FD}}}\frac{\partial {p}_{{\rm{FD}}}}{\partial {t}_{{\rm{D}}}}\\ {\eta }_{{\rm{MD}}}\frac{\partial {p}_{{\rm{MD}}}}{\partial {t}_{{\rm{D}}}}={\lambda }_{{\rm{FM}}}({p}_{{\rm{FD}}}-{p}_{{\rm{MD}}});\,{\eta }_{{\rm{vD}}}\frac{\partial {p}_{{\rm{vD}}}}{\partial {t}_{{\rm{D}}}}={\lambda }_{{\rm{Fv}}}({p}_{{\rm{FD}}}-{p}_{{\rm{vD}}})\\ {p}_{{\rm{FD}}}({r}_{{\rm{D}}},{t}_{{\rm{D}}}=0)={p}_{{\rm{MD}}}({r}_{{\rm{D}}},{t}_{{\rm{D}}}=0)={p}_{{\rm{vD}}}({r}_{{\rm{D}}},{t}_{{\rm{D}}}=0)=0\\ {p}_{{\rm{FD}}}({r}_{{\rm{D}}}={r}_{{\rm{eD}}},{t}_{{\rm{D}}})=0;\,{\rm{or}}\,{p}_{{\rm{FD}}}({r}_{{\rm{D}}}={r}_{{\rm{eD}}}\to \infty ,{t}_{{\rm{D}}})=0;\,{\rm{or}}\,{\frac{\partial {p}_{{\rm{FD}}}}{\partial {r}_{{\rm{D}}}}|}_{{r}_{{\rm{D}}}={r}_{{\rm{eD}}}}=0\\ {\frac{\partial {p}_{{\rm{1D}}}}{\partial {r}_{{\rm{D}}}}|}_{{r}_{{\rm{D}}}=1}=-\frac{1}{{M}_{1}};\,{{p}_{{\rm{1D}}}|}_{{r}_{{\rm{D}}}={r}_{{\rm{cD}}}}={{p}_{{\rm{FD}}}|}_{{r}_{{\rm{D}}}={r}_{{\rm{cD}}}};\,{M}_{{\rm{1}}}{r}_{{\rm{cD}}}^{{d}_{{\rm{f}}}-\theta -2}{\frac{\partial {p}_{{\rm{1D}}}}{\partial {r}_{{\rm{D}}}}|}_{{r}_{{\rm{D}}}={r}_{{\rm{cD}}}}={\frac{\partial {p}_{{\rm{FD}}}}{\partial {r}_{{\rm{D}}}}|}_{{r}_{{\rm{D}}}={r}_{{\rm{cD}}}}\end{array}$$

**Table 1:** Summary of the variables in the model.

Defined parameters	Expressions
Dimensionless time, *t*_D_	${t}_{{\rm{D}}}={k}_{{\rm{F}}}t/[({\phi }_{{\rm{M}}}{C}_{{\rm{M}}}+{\phi }_{{\rm{F}}}{C}_{{\rm{F}}}+{\phi }_{{\rm{v}}}{C}_{{\rm{v}}})\mu {r}_{{\rm{w}}}^{2}]$
Dimensionless pressure, *p*_D_	${p}_{{\rm{D}}}=2{\rm{\pi }}{k}_{{\rm{F}}}h({p}_{{\rm{0}}}-p)/(qB\mu )$
Dimensionless radius, *r*_D_	${r}_{{\rm{D}}}=r/{r}_{{\rm{w}}}$
Diffusivity ratio between the two regions, *η*_1D_	${\eta }_{{\rm{1D}}}={C}_{{\rm{t1}}}{\phi }_{1}/({\phi }_{{\rm{M}}}{C}_{{\rm{M}}}+{\phi }_{{\rm{F}}}{C}_{{\rm{F}}}+{\phi }_{{\rm{v}}}{C}_{{\rm{v}}})$
Fracture storage capacitance coefficient, *η*_FD_	${\eta }_{{\rm{FD}}}={C}_{{\rm{F}}}{\phi }_{{\rm{F}}}/({\phi }_{{\rm{M}}}{C}_{{\rm{M}}}+{\phi }_{{\rm{F}}}{C}_{{\rm{F}}}+{\phi }_{{\rm{v}}}{C}_{{\rm{v}}})$
Matrix storage capacitance coefficient, *η*_MD_	${\eta }_{{\rm{MD}}}={C}_{{\rm{M}}}{\phi }_{{\rm{M}}}/({\phi }_{{\rm{M}}}{C}_{{\rm{M}}}+{\phi }_{{\rm{F}}}{C}_{{\rm{F}}}+{\phi }_{{\rm{v}}}{C}_{{\rm{v}}})$
Vug storage capacitance coefficient, *η*_vD_	${\eta }_{{\rm{vD}}}={C}_{{\rm{v}}}{\phi }_{{\rm{v}}}/({\phi }_{{\rm{M}}}{C}_{{\rm{M}}}+{\phi }_{{\rm{F}}}{C}_{{\rm{F}}}+{\phi }_{{\rm{v}}}{C}_{{\rm{v}}})$
Permeability ratio, *M*_1_	${M}_{1}={k}_{{\rm{1}}}/{k}_{{\rm{F}}}$
Fracture-matrix inter-porosity coefficient, *λ*_FM_	${\lambda }_{{\rm{FM}}}={\alpha }_{{\rm{FM}}}{r}_{{\rm{w}}}^{2}{k}_{{\rm{M}}}/{k}_{{\rm{F}}}$
Fracture-vug inter-porosity coefficient, *λ*_Fv_	${\lambda }_{{\rm{Fv}}}={\alpha }_{{\rm{Fv}}}{r}_{{\rm{w}}}^{2}{k}_{{\rm{v}}}/{k}_{{\rm{F}}}$

Based on the derived process stated in Appendix B, the analytical solution for Eq. (8) in Laplace space can be obtained. The wellbore pressure in Laplace space considering the wellbore storage and the skin effects is

9 $${\bar{p}}_{{\rm{wD}}}=\frac{z[A{I}_{n}(\beta )+B{K}_{n}(\beta )]+S}{z[1+{C}_{{\rm{D}}}z(z[A{I}_{n}(\beta )+B{K}_{n}(\beta )]+S)]}$$

where *S* is the skin factor, *C*_D_ is the dimensionless wellbore storage coefficient. Then, with the Stehfest inversion algorithm^51^, the wellbore pressure in real space can be obtained.

## Results

### Validation of the proposed model

To validate the derived analytical solution, the dimensionless pressure and derivative (*P*_wD_ and d*P*_wD_) of the new derived model are compared with numerical solutions. In the calculation, the outer boundary condition is set as constant pressure, and the function *g*(*z*) in Eq. (11) is set to be 1 (the outer region of the model can be simplified as the homogeneous porous media). Table 2 summarizes the data used for the comparison. Result (Fig. 3) illustrates that calculations from the proposed model agree well with those from numerical simulation.

**Table 2:** Synthetic data used for validation.

Cases	Parameters							
	*r*_eD_	*r*_cD_	*C*_D_	*S*	*d*_f_	*θ*	*η*_1D_	*M*_1_
1	7460	260	100	0	2	0	1	1
2	7460	260	100	0	2	0	1	0.05
3	7460	260	100	0	1.75	0	1	0.05
4	7460	260	100	0	1.75	0.25	3	10

### Comparison with former models

**Figure 4:**
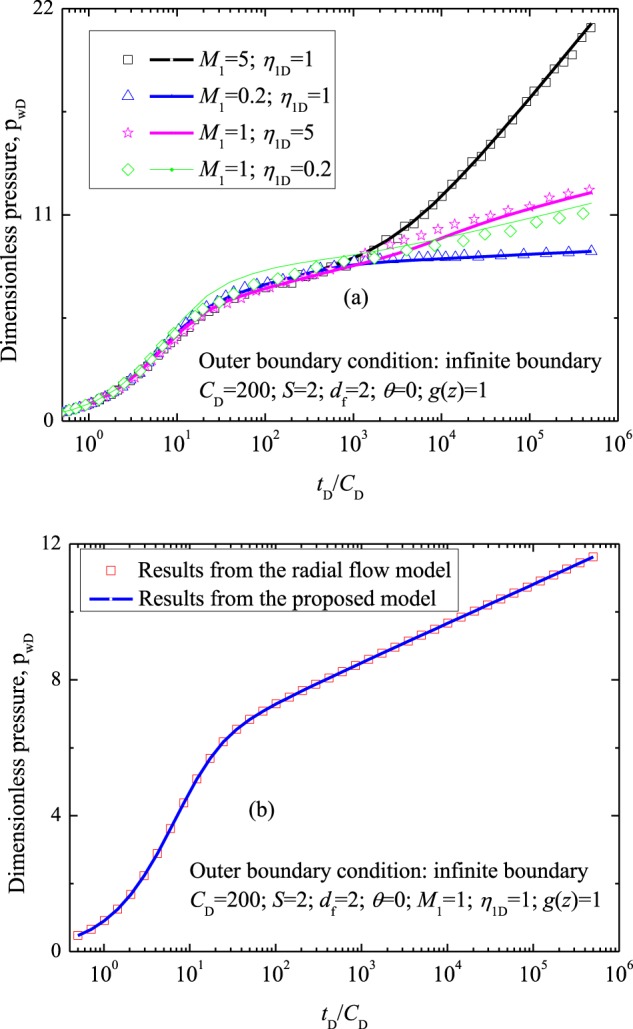
Comparison of *P*_wD_ obtained from the proposed model and other models: (**a**) for radial composite model; (**b**) for radial model. Results from former models are shown with points, and calculations from the derived model are solid lines.

When parameters *d*_f_, *θ* and *g*(*z*) in our derived model are assigned as 2, 0 and 1, respectively, the acidized region (inner region) can be considered homogeneous, and the reservoir can be regarded as two different but uniform regions. In this case, our derived model can be validated with radial composite model. Figure 4a presents the comparison of the results (*P*_wD_ versus *t*_D_) from the derived model and that from fractal radial composite model proposed by Razminia *et al*.^52^ When parameters *d*_f_ and *θ* in fractal radial composite model are set to be 2 and 0, respectively, fractal radial composite model can be simplified as the conventional radial composite model. In the calculation, the outer boundary condition is set as infinite boundary, other parameters applied in the model assigned were: *C*_D_ = 200, *S* = 2 and *r*_cD_ = 500. Results (Fig. 4a) suggest that the derived model is validated. When parameters *d*_f_, *θ*, *M*_1_, *η*_1D_, and *g*(*z*) in our derived model are assigned as 2, 0, 1, 1 and 1, respectively, the reservoir can be regarded as a homogeneous porous media. In this case, the derived model can be compared with the classic radial flow model derived by Agarwal *et al*.^53^ Fig. 4b presents the comparison of the results (*P*_wD_ versus *t*_D_) from the derived model and that from the classic analytical solution^53^. In the calculation, the outer boundary condition is set as infinite boundary, other parameters applied in the model assigned were: *C*_D_ = 200 and *S* = 2. Results (Fig. 4b) also suggest that the derived model is validated. Figure 4a,b show the former classic models are special cases of the new derived model, and the new derived analytical model can be used to analyze more complex flow behaviors.

### Flow characteristic curves

**Figure 5:**
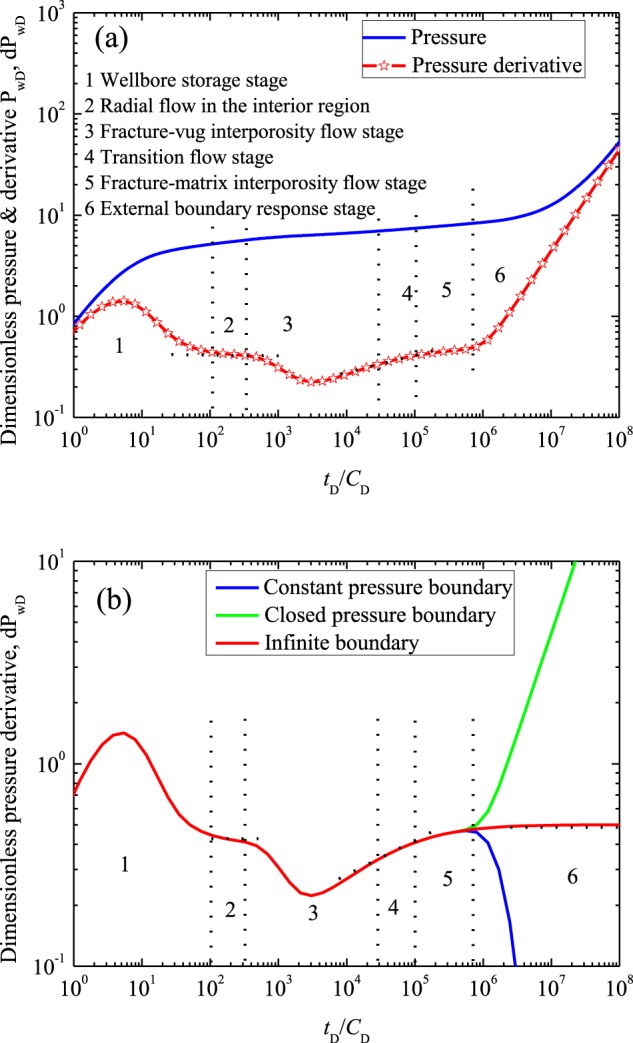
Flow characteristic curves: (**a**) pressure & pressure derivative curves for closed boundary condition; (**b**) pressure derivative curves for three different outer boundary conditions.

As stated in the literatures^5,33^, the flow characteristic curves can be used for recognizing flow behavior in the reservoir. Taking the closed outer boundary condition as example, the flow characteristic curve will be discussed in detail. Figure 5a presents the log-log curves (i.e., *t*_D_ versus *P*_wD_ and d*P*_wD_) for closed outer boundary condition. In the calculation, fractal dimension of the pore spaces *d*_f_ is 2, the conductivity index *θ* is 0, the dimensionless parameters *r*_eD_ and *r*_cD_ are 30000 and 2000, respectively, skin factor *S* assigned is 0, *C*_D_ assigned is 200, diffusivity ratio between the two regions *η*_1D_ is 0.127, fracture storage capacitance coefficient *η*_FD_ is 0.022, matrix storage capacitance coefficient *η*_MD_ is 0.106, vug storage capacitance coefficient *η*_vD_ is 0.872, permeability ratio *M*_1_ is 1.251, *λ*_FM_ assigned is 2.01 × 10^−7^, and *λ*_Fv_ assigned is 9.70 × 10^−3^. With the same parameters, Fig. 5b compares the differences of three well testing curves to study the effect of outer boundary on flow characteristics. Results (Fig. 5a,b) illustrate that the flow characteristic curves can be characterized by six flow stages:

The first stage is well storage stage, in which the fluid flow is dominated by the parameters *S* and *C*_D_.

The second stage is radial flow in the inner region. The d*P*_wD_ curve shows zero slope horizontal line. During this period, the fluid in the interior region flows into the wellbore. The flow characteristic is mainly controlled by the parameters *r*_cD_, pore spaces fractal dimension and the conductivity index. The larger the range of the inner region is, the longer the stage 2 will be.

The third stage is fracture-vug inter-porosity flow stage, in which the d*P*_wD_ curve shows the first concave. The concave shape represents the supplement between the fracture system and vug system. In this stage, the pressure wave has propagated to the coupling interface, and the flow characteristic in this period is mainly controlled by fracture-vug inter-porosity coefficient.

The fourth stage is transition flow stage, which presents a straight line with the slope 0.5 on the log-log curve of *t*_D_ versus d*P*_wD_.

The fifth stage is fracture-matrix inter-porosity flow stage, in which the d*P*_wD_ curve shows the second concave shape. The second concave shape segment reflects supplement from matrix to fracture. During this stage, flow characteristic is mainly controlled by fracture-matrix inter-porosity coefficient.

The sixth stage is external boundary response stage, in which flow characteristic in the reservoir is mainly controlled by the parameter *r*_eD_.

### Sensitivity analysis

In this part, we will study the influences of parameters on well testing curves in detail. The outer boundary is set as closed pressure, the parameter values is listed in Table 3. The presented model demonstrates that *d*_f_, *θ*, *r*_c_, *L*_M_, *L*_F_ and *L*_v_ are the basic parameters, which can determine the flow characteristic in acidized fractured-vuggy reservoirs. The basic parameters and their ranges used for sensitivity analysis are summarized in Table 4.

**Table 3:** Parameter values.

Parameter	Parameter value	Parameter	Parameter value
*φ*_M_	0.1	*r*_eD_	30000
*r*_w_/m	0.1	*r*_cD_	2000
*L*_M_/m	2	*η*_1D_	0.127
*L*_F_/m	8 × 10^−4^	*η*_FD_	0.022
*L*_v_/m	1.08	*η*_MD_	0.106
*k*_1_/m^2^	1.0 × 10^−10^	*η*_vD_	0.872
*K*_M_/m^2^	1.6 × 10^−14^	*λ*_FM_	2.01 × 10^−7^
*d*_f_	2	*λ*_Fv_	9.66 × 10^−3^
*θ*	0	*M*_1_	1.56

**Table 4:** Ranges of parameters for sensitivity analysis.

Parameter	Range of the parameter	Parameter	Range of the parameter
*d*_f_	1.65 ≤ *d*_f_ ≤ 2	*L*_M_/m	1.5 ≤ *L*_M_ ≤ 2.5
*θ*	−0.25 ≤ *θ* ≤ 0.25	*L*_F_/m	3 × 10^−4^ ≤ *L*_F_ ≤ 8 × 10^−4^
*r*_c_/m	100 ≤ *r*_c_ ≤ 300	*L*_v_/m	0.8 ≤ *L*_v_ ≤ 1.2

**Figure 6:**
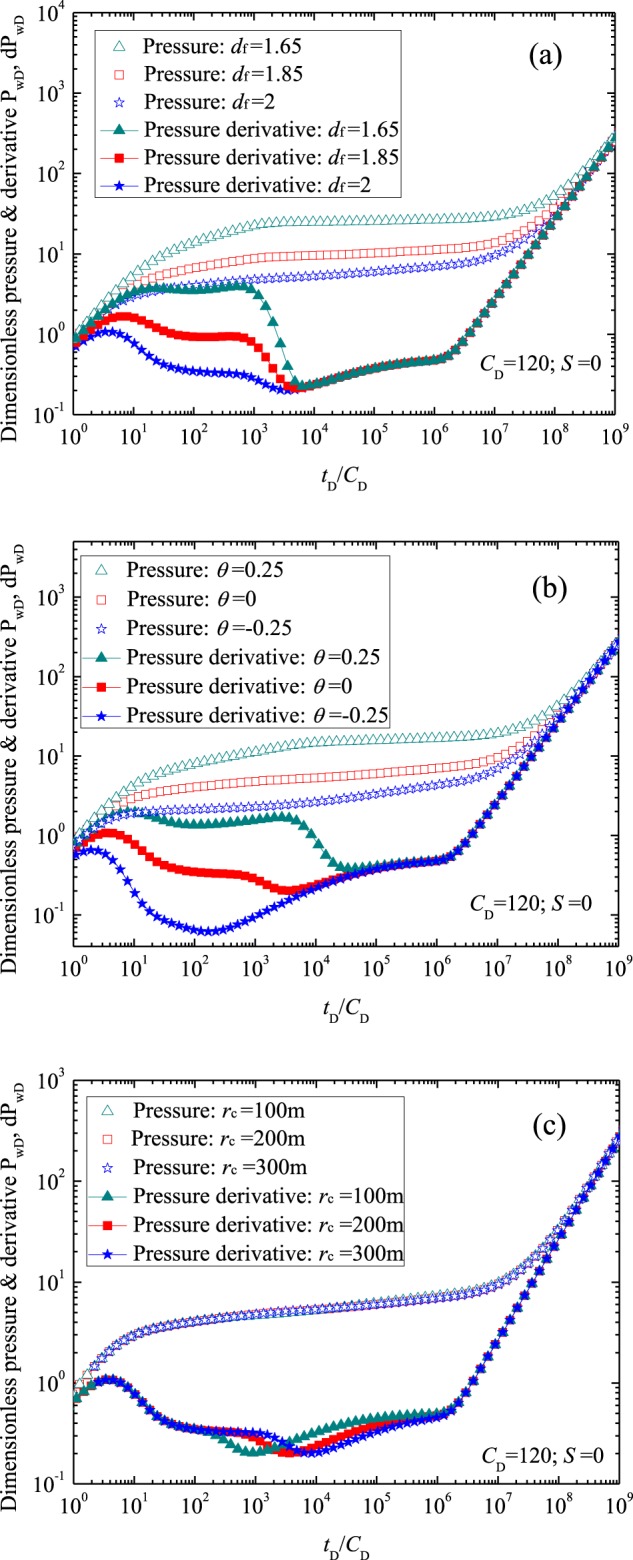
Effects of parameters of inner region on the well testing curves: (**a**) for inner region fractal dimension; (**b**) for inner region connectivity index; (**c**) for parameter *r*_c_.

Figure 6 shows flow characteristic curves with different inner region parameters (i.e., inner region fractal dimension, inner region connectivity index and inner region radius). Figure 6a presents the effect of parameter *d*_f_ on the flow characteristic curves (for 1.65 ≤ *d*_f_ ≤ 2). As shown in Fig. 6a, *P*_wD_ and d*P*_wD_ in stages 1, 2 and 3 decrease with the increased inner region fractal dimension *d*_f_. This can be interpreted as that the larger inner region fractal dimension *d*_f_ implies inner region permeability increasing, leading to the decrease of flow resistance and the dimensionless pressure. Figure 6b demonstrates the effect of inner region connectivity index *θ* on the flow characteristic curves (for −0.25 ≤ *θ* ≤ 0.25). Figure 6b demonstrates *P*_wD_ and d*P*_wD_ in stages 1, 2 and 3 increase with the increased inner region connectivity index *θ*. Figure 6c illustrates the effect of inner region radius on the flow characteristic curves (for 100 m ≤ *r*_c_ ≤ 300 m). Results (Fig. 6c) show *P*_wD_ and d*P*_wD_ in stages 3 to 5 decrease with the increased inner region radius. The main reason for this is the permeability at the edge of the wellbore *k*_1_ is larger than fracture system permeability *k*_F_ (*M*_1_ > 1). Figure 6c also shows a longer period of radial flow (stage 2) corresponds to a larger inner region radius, which is expected. Figure 6 demonstrates that forming larger acidized region can help improve well production and reduce fluid pressure loss.

**Figure 7:**
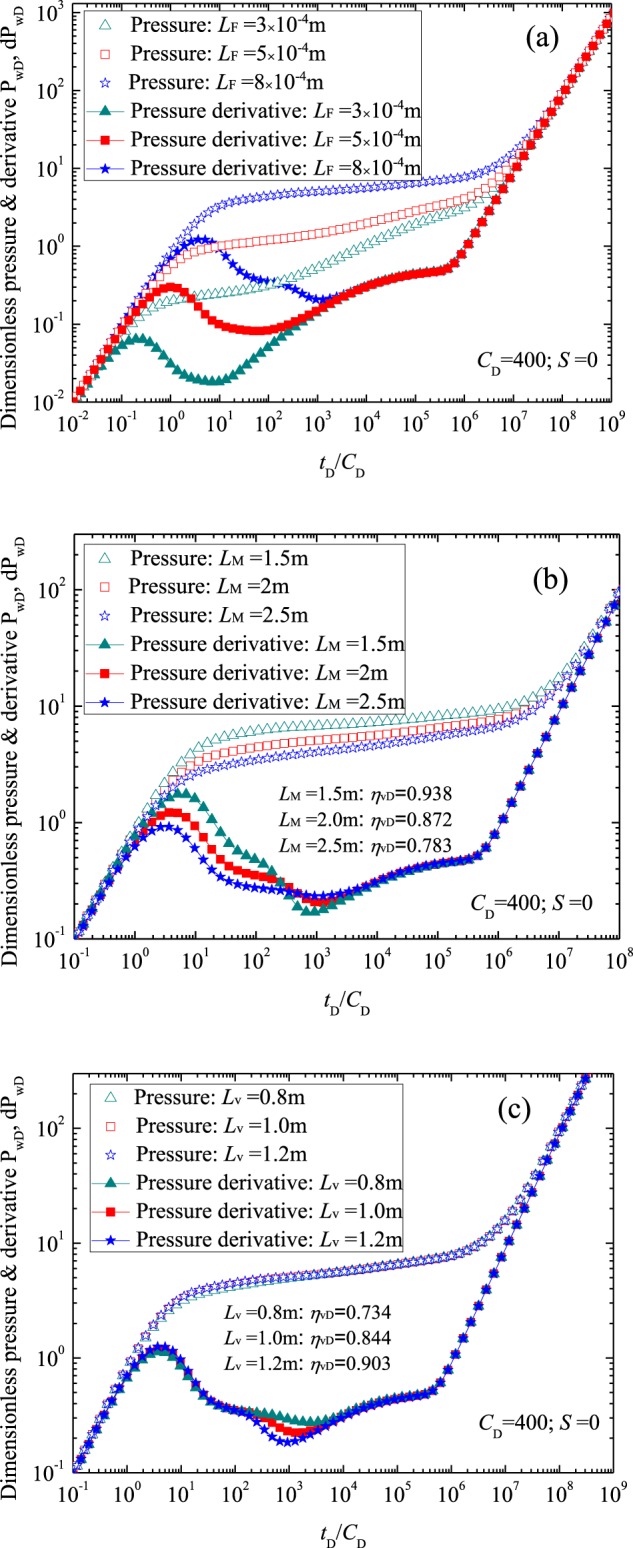
Effect of basic parameters in outer region on the dimensionless wellbore pressure: (**a**) for the space interval of fracture in outer region; (**b**) for the matrix block size in outer region; (**c**) for the radius of the vug in outer region.

Figure 7 presents the influences of the outer region parameters, such as the space interval of fracture system *L*_F_, matrix block size *L*_M_ and radius of the vug *L*_v_, on the transient pressure behavior. Figure 7a presents the effect of the space interval of fracture system *L*_F_ on the flow characteristic curves (for 3 × 10^−4^ m ≤ *L*_F_ ≤ 8 × 10^−4^ m). As shown in Fig. 7a, a larger *L*_F_ corresponds to a larger pressure depletion. The main reason is that a greater parameter *L*_F_ value results in a smaller parameter *M*_1_ and the smaller parameter *η*_1D_. We can see that the effect of parameter *L*_F_ on the well testing curves is concentrated in stages 1 to 4. Figure 7b demonstrates the effect of matrix block size in outer region on the flow characteristic curves (for 1.5 m ≤ *L*_M_ ≤ 2.5 m). We can see that *P*_wD_ in stages 1 to 4 decreases with the increasing matrix block size *L*_M_. As a larger *L*_M_ corresponding to a larger *M*_1_, which leads to a smaller pressure depletion in stages 1 and 2. Furthermore, parameter *η*_vD_ decreases as parameter *L*_M_ increases, indicating the supplement of the vug system to fracture system decreases will the increasing parameter *L*_M_. As a result, the dimensionless pressure derivation in stage 3 increases with the increasing *L*_M_. Figure 7c shows the effect of parameter *L*_v_ in outer region on the flow characteristic curves (for 0.5 m ≤ *L*_v_ ≤ 1.2 m). It can be seen that the effects of *L*_v_ on *P*_wD_ and d*P*_wD_ curves are concentrated in stage 3, where a larger *L*_v_ will lead to a smaller pressure depletion. The main reason is that, a larger *L*_v_ implies larger energy in the vugs, leading to increased supply from the vug system to the fracture system, which makes the pressure derivative values smaller.

### Case example

**Figure 8:**
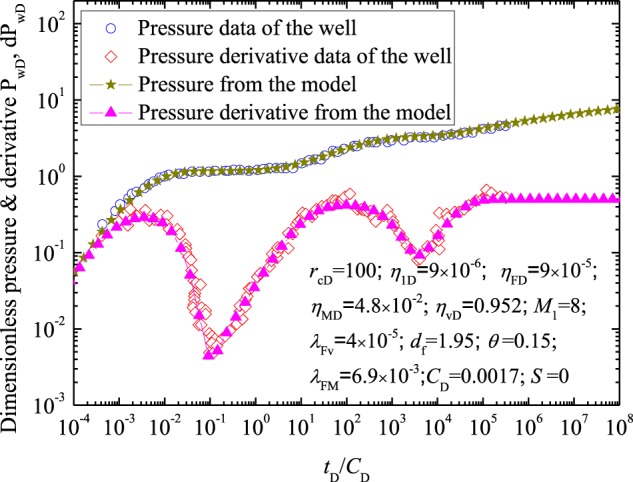
Comparison of the calculated results with actual test data.

In this case, pressure transient data from an acidized well in Tahe oilfield (Tarim basin, China), is selected to interpret. The actual test data of the acidized well are cited from Wang *et al*.^33^. In the calculation, the parameters measured are *P*_wD_ and *d*P_wD_ and the corresponding *t*_D_, and the fitting parameters mainly concludes: *d*_f_; *θ*; *r*_cD_; *η*_1D_; *η*_FD_; *η*_MD_; *η*_vD_; *M*_1_; *λ*_Fv_; *λ*_FM_; *C*_D_ and *S*. Figure 8 presents the fitting results and the actual test data, and the corresponding results by well test interpretation are as follows: *d*_f_ = 1.95; *θ* = 0.15; *r*_cD_ = 100; *η*_1D_ = 9 × 10^−6^; *η*_FD_ = 9 × 10^−5^; *η*_MD_ = 4.8 × 10^−2^; *η*_vD_ = 0.952; *M*_1_ = 8; *λ*_Fv_ = 4 × 10^−5^; *λ*_FM_ = 6.9 × 10^−3^; *C*_D_ = 0.0017; *S* = 0. Figure 8 illustrates that the results agree with each other, which verifies the presented model.

## Discussions

### Advantages and limitations of the derived model

The analytical model lays theoretical foundations for characterizing the flow characteristic curves and dynamic pressure of acidized well in acidized carbonate porous media. With this new analytical solution, it can help to reduce the uncertainty in flow through porous media and obtain data with high accuracy. In addition, it can be applied to field case study and estimate more accurate reservoir parameters reflecting flow characteristic using inverse modeling. However, it should be noted that the derived model is limited to single-phase flow. Multi-phase fluid flow in fractured-vuggy carbonate porous medium is an interesting and challenging topic, and the corresponding work is still under processing.

## Conclusions

Based on fractal theory and triple medium concept model, a novel analytical solution to a fractal composite model is derived to study the flow characteristic in acidized carbonate reservoirs. Predicted results deduced from the derived analytical solution agree well with numerical simulation results. For the closed outer boundary condition, the influence of basic parameters, such as *d*_f_, *θ*, *r*_c_, *L*_M_, *L*_F_ and *L*_v_, on flow characteristic curves are studied in detail. Followings are the main conclusions:

1. The calculated results (both *P*_wD_ and d *P*_wD_) based on the derived analytical solution match the actual test data very well, which illustrates this proposed model can be applied to field case study.
2. As the inner region permeability is larger than fracture system permeability, forming larger acidized region can help improve well production and reduce fluid pressure loss.
3. The new analytical solution to the fractal composite model, considering the heterogeneity of the pore networks in acidized region, is useful for flow characteristic analysis of acidized well in acidized carbonate porous media. With this new analytical solution, it can help to reduce the uncertainty in flow through porous media and obtain data with high accuracy. In addition, it can be applied to estimate more accurate reservoir parameters reflecting flow characteristic using inverse modeling.

## Supplementary information

Appendix

## References

[1] Kossack, C. A., Gurpinar, O. A methodology for simulation of vuggy and fractured reservoirs. Paper SPE-66366-MS presented at the SPE Reservoir Simulation Symposium, Houston, Texas, 11–14 February (2001).

[2] Rivas-Gomez, S. *et al*. Numerical simulation of oil displacement by water in a vuggy fractured porous medium. Paper SPE-66386-MS presented at SPE Reservoir Simulation Symposium, Houston, Texas, 11–14 February (2001).

[3] Camacho-Velazquez, R. *et al*. Pressure transient and decline curve behaviors in naturally fractured vuggy carbonate reservoirs. Paper SPE-77689-MS presented at SPE Annual Technical Conference and Exhibition, San Antonio, Texas, 29 September-2 October (2002).

[4] Kang ZJ (2005). Percolation characteristics of fractured-vuggy carbonate reservoir in Tahe oilfield. Oil Gas Geol..

[5] Gao B (2016). Pressure transient analysis of a well penetrating a filled cavity in naturally fractured carbonate reservoirs. Journal of Petroleum Science and Engineering.

[6] Lyu X (2017). Mechanism and influencing factors of EOR by N2 injection in fractured-vuggy carbonate reservoirs. Journal of Natural Gas Science and Engineering.

[7] Fuentes-Cruz, G., Camacho-Velazquez, R. & Vásquez-Cruz, M. Pressure transient and decline curve behaviors for partially penetrating wells completed in naturally fractured-vuggy reservoirs. Paper SPE-92116-MS presented at SPE International Petroleum Conference in Mexico, 7–9 November (2004).

[8] Jia YL (2013). Flow modeling of well test analysis for porous–vuggy carbonate reservoirs. Transport in Porous Media.

[9] Guo JC, Nie RS, Jia YL (2012). Dual permeability flow behavior for modeling horizontal well production in fractured-vuggy carbonate reservoirs. Journal of hydrology.

[10] Popov P (2009). Multiphysics and multiscale methods for modeling fluid flow through naturally fractured carbonate karst reservoirs. SPE Reservoir Evaluation & Engineering.

[11] Rzonca B (2008). Carbonate aquifers with hydraulically non-active matrix: a case study from Poland. Journal of hydrology.

[12] Huang, T. P., Hill, A. D. & Schechter, R. S. Reaction rate and fluid loss: the key to wormhole initiation and propagation in carbonate acidizing. Presented at International Symposium on Oilfield Chemistry, 18–21 February, Houston, Texas, SPE-37312-MS (1997).

[13] Qiu, X. *et al*. Quantitative modeling of acid wormholing in carbonates-what are the gaps to bridge. Paper SPE-164245-MS presented at SPE Middle East Oil and Gas Show and Conference (2013).

[14] Kang Q (2002). Lattice Boltzmann simulation of chemical dissolution in porous media. Physical Review E.

[15] Mostofizadeh, B. & Economides, M. J. Optimum injection rate from radial acidizing experiments. Paper SPE-28547-MS presented at SPE Annual Technical Conference and Exhibition, New Orleans, Louisiana, 25–28 September (1994).

[16] Frick, T. P., Mostofizadeh, B. & Economides, M. J. Analysis of radial core experiments for hydrochloric acid interaction with limestones. Paper SPE-27402-MS presented at SPE Formation Damage Control Symposium, Lafayette, Louisiana, 7–10 February (1994).

[17] Fredd, C. N. Dynamic model of wormhole formation demonstrates conditions for effective skin reduction during carbonate matrix acidizing. Paper SPE-59537-MS presented at SPE Permian Basin Oil and Gas Recovery Conference, Midland, 21–23 March (2000).

[18] Tremblay B (2005). Modelling of sand transport through wormholes. Journal of Canadian Petroleum Technology.

[19] Liu M (2013). Wormhole propagation behavior under reservoir condition in carbonate acidizing. Transport in Porous Media.

[20] Zakaria Mohamed Reda A. Tracer fluid flow through porous media: theory applied to acid stimulation treatments in carbonate rocks. PhD thesis, Texas A. & M. University (2014).

[21] Li, X. *et al*. Large-scale visual experiment and numerical simulation of acid fingering during carbonate acid fracturing. Paper SPE-187019-MS presented at SPE/IATMI Asia Pacific Oil & Gas Conference and Exhibition, Jakarta, Indonesia, 17–19 October (2017).

[22] Dejam M, Hassanzadeh H, Chen Z (2014). Shear dispersion in a fracture with porous walls. Advances in water resources.

[23] Dejam, M., Hassanzadeh, H. & Chen, Z. Shear dispersion in a capillary tube with a porous wall. *Journal of contaminant hydrology*, 2016, **185**, 87–104 (2016). 10.1016/j.jconhyd.2016.01.007 26845232

[24] Dejam M, Hassanzadeh H, Chen Z (2018). A reduced-order model for chemical species transport in a tube with a constant wall concentration. The Canadian Journal of Chemical Engineering.

[25] Dejam M (2018). Dispersion in non-Newtonian fluid flows in a conduit with porous walls. Chemical Engineering Science.

[26] Dejam M, Hassanzadeh H, Chen Z (2018). Shear dispersion in a rough-walled fracture. SPE Journal.

[27] Dejam M (2019). Advective-diffusive-reactive solute transport due to non-Newtonian fluid flows in a fracture surrounded by a tight porous medium. International Journal of Heat and Mass Transfer.

[28] Ugursal, A. Development of acid fracturing model for naturally fractured reservoirs. PhD thesis, Texas A&M University (2018).

[29] Wu, Y. S. *et al*. A multiple-continuum approach for modeling multiphase flow in naturally fractured vuggy petroleum reservoirs. Paper SPE-104173-MS presented at International Oil & Gas Conference and Exhibition in China, 5–7 December, Beijing, China (2006).

[30] Wu, Y. S. *et al*. A triple-continuum pressure-transient model for naturally fractured vuggy reservoir. Paper SPE-110044-MS presented at SPE Annual Technical Conference and Exhibition, Anaheim, 11–14 Nov. (2007).

[31] Wu YS, Qin G (2009). A generalized numerical approach for modeling multiphase flow and transport in fractured porous media. Communications in computational physics.

[32] Yao, J., Huang, Z. Q. & Li, Y. Z. Discrete fracture-vug network model for modeling fluid flow in fractured vuggy porous media. Paper SPE-130287-MS presented at International Oil and Gas Conference and Exhibition in China, Beijing, 8–10 June (2010).

[33] Wang L (2014). Analytical modeling of flow behavior for wormholes in naturally fractured-vuggy porous media. Transport in porous media.

[34] Wang L, Chen X, Xia Z (2018). A novel semi-analytical model for multi-branched fractures in naturally fractured-vuggy reservoirs. Scientific reports.

[35] Yang J, Yao J, Wang Z (2005). Study of pressure transient characteristic for triple-medium composite reservoirs. Journal of Hydrodynamics.

[36] Chen F (2008). The flow model of the triple-medium composite reservoirs and the type curves. Journal of Daqing Petroleum Institute.

[37] Yao YD, Wu YS, Zhang RL (2012). The transient flow analysis of fluid in a fractal, double-porosity reservoir. Transport in porous media.

[38] Liu P (2015). Analysis and simulation of rheological behavior and diverting mechanism of in situ self-diverting acid. Journal of Petroleum Science and Engineering.

[39] Zhang HW (2017). Dual fractal model of carbonate acidizing wormholes. Natural Gas Geoscience.

[40] Fredd, C. N. & Miller, M. J. Validation of carbonate matrix stimulation models. Paper SPE-58713-MS presented at SPE International Symposium on Formation Damage Control, Lafayette, Louisiana 23–24 February (2000).

[41] Liu M, Zhang SC, Mou J (2012). Dissolution pattern of radial wormhole model in carbonate acidizing. Petroleum Geology and Recovery Efficiency.

[42] Abdassah D, Ershaghi I (1986). Triple-porosity systems for representing naturally fractured reservoirs. SPE Formation Evaluation.

[43] Li XG, Yang ZZ, Su JZ (2010). Fractal mathematical model of 3D acid corrosion of rough carbonate rock particles. Xingjiang Petroleum Geology.

[44] Liu M (2012). Fractal nature of acid-etched wormholes and the influence of acid type on wormholes. Petroleum Exploration and Development.

[45] Zhang HW (2017). Fractal model of carbonate acidizing wormhole and acidizing parameter optimization. Journal of Southwest Petroleum University (Science & Technology Edition).

[46] Di Y (2013). A method to determine the size limit between dissolution pores and vugs in triple medium. Journal of Basic Science and Engineering.

[47] Acuna JA, Ershaghi I, Yortsos YC (1995). Practical application of fractal pressure transient analysis of naturally fractured reservoirs. SPE Formation Evaluation.

[48] Wang W, Shahvali M, Su Y (2015). A semi-analytical fractal model for production from tight oil reservoirs with hydraulically fractured horizontal wells. Fuel.

[49] Cossio M, Moridis G, Blasingame TA (2013). A semianalytic solution for flow in finite-conductivity vertical fractures by use of fractal theory. SPE Journal.

[50] Chang J, Yortsos YC (1990). Pressure transient analysis of fractal reservoirs. SPE Formation Evaluation.

[51] Stehfest H (1970). Algorithm 368: numerical inversion of laplace transforms [D5]. Communications of the ACM.

[52] Razminia K, Razminia A, Trujilo JJ (2015). Analysis of radial composite systems based on fractal theory and fractional calculus. Signal Processing.

[53] Agarwal RG, Al-Hussainy R, Ramey HJ (1970). An investigation of wellbore storage and skin effect in unsteady liquid flow: I. Analytical treatment. Society of Petroleum Engineers Journal.

