# Supplementary material for: A new analytical model for flow in acidized fractured-vuggy porous media
Source: Sci Rep. 2019 Jun 5;9:8293. doi: 10.1038/s41598-019-44802-2 (PMC6549157; doi:10.1038/s41598-019-44802-2)
Supplement: Supplementary file 1 — Appendix [file 41598_2019_44802_MOESM1_ESM.pdf]

A new analytical model for flow in acidized fractured-vuggy porous media

Gang Lei[1], Qinzhuo Liao[1]*, Dongxiao Zhang[2]

[1]Department of Petroleum Engineering, CPG, King Fahd University of Petroleum and Minerals, Dhahran, Saudi Arabia

[2]ERE & BIC-ESAT, College of Engineering, Peking University, Beijing, China

*Corresponding author. Email: qinzhuo.liao@kfupm.edu.sa

Appendix A. Theoretical model for physical parameters of triple medium

According to the fractured-vuggy conceptual model for triple medium shown in Figure 4, the fracture system porosity and vug system porosity for triple medium can be respectively obtained as[45]

$$\varphi_{\mathrm{F}} = 1 - \frac{L_{\mathrm{M}}^{3}}{\left(L_{\mathrm{M}} + L_{\mathrm{F}}\right)^{3}} \tag{A1}$$

$$\varphi_{\mathrm{v}} = \frac{\pi L_{\mathrm{v}}^{3}}{6\left(L_{\mathrm{M}} + L_{\mathrm{F}}\right)^{3}} \tag{A2}$$

Where φ_{F} is the porosity for fracture system, φ_{v} is the porosity for vug system, L_{M} is the matrix block size, L_{F} and L_{v} are the space interval of fracture and radius of the vug, respectively.

Assuming fracture system can be considered as the main pathway of global flow and vug system permeability is equal to the permeability of the surrounding fracture system, then, vug permeability is

$$K_{\mathrm{v}} = \frac{L_{\mathrm{F}}^{2}}{12} \tag{A3}$$

When the effect of fracture system porosity is considered, fracture permeability is

$$K_{\mathrm{F}} = \frac{\varphi_{\mathrm{F}} L_{\mathrm{F}}^{2}}{12} = \left[1 - \frac{L_{\mathrm{M}}^{3}}{\left(L_{\mathrm{M}} + L_{\mathrm{F}}\right)^{3}}\right]\frac{L_{\mathrm{F}}^{2}}{12} \tag{A4}$$

where K_v is vug system permeability, and K_F is the permeability of fracture system.

As stated in the literature, the shape factors for fracture-vug and fracture-matrix can be respectively expressed as[45]

$$\alpha_{Fv} = \sqrt[3]{\frac{4\pi}{3}}\frac{3\sqrt{2}\pi L_v L_F}{4\left(L_M + L_F\right)^4} \tag{A5}$$

$$\alpha_{FM} = \sqrt[3]{\frac{4\pi}{3}}\frac{4\varphi_M L_M^2}{\left(2L_M + L_F\right)\left(L_M + L_F\right)^3} \tag{A6}$$

Appendix B. Analytical solution of Eq. (8) in Laplace space

Through the Laplace transform,

$$\overline{p}_D\left(r_D, z\right) = \int_0^{\infty} e^{-zt_D}\,\overline{p}_D\left(r_D, t_D\right) dt_D \tag{B1}$$

Eq. (8) can be rewritten as

$$\begin{cases} \dfrac{d^2\overline{p}_{1D}}{dr_D^2} + \dfrac{d_f - \theta - 1}{r_D}\dfrac{d\overline{p}_{1D}}{dr_D} = \dfrac{\eta_{1D}}{M_1} r_D^{\theta} z\overline{p}_{1D} \\ \dfrac{1}{r_D}\dfrac{d}{dr_D}\left(r_D\dfrac{d\overline{p}_{FD}}{dr_D}\right) = g\left(z\right) z\overline{p}_{FD} \\ \overline{p}_{FD}\left(r_D = r_{eD}, t_D\right) = 0; \quad \text{or } \overline{p}_{FD}\left(r_D = r_{eD} \to \infty, t_D\right) = 0; \quad \text{or } \left.\dfrac{d\overline{p}_{FD}}{dr_D}\right|_{r_D = r_{eD}} = 0 \\ \left.\dfrac{dp_{1D}}{dr_D}\right|_{r_D = 1} = -\dfrac{1}{zM_1}; \quad \left.\overline{p}_{1D}\right|_{r_D = r_{cD}} = \left.\overline{p}_{FD}\right|_{r_D = r_{cD}}; \quad M_1 r_{cD}^{d_f - \theta - 2}\left.\dfrac{d\overline{p}_{1D}}{dr_D}\right|_{r_D = r_{cD}} = \left.\dfrac{d\overline{p}_{FD}}{dr_D}\right|_{r_D = r_{cD}} \end{cases} \tag{B2}$$

where

$$g\left(z\right) = \frac{\eta_{MD}\lambda_{FM}}{\eta_{MD}z + \lambda_{FM}} + \frac{\eta_{vD}\lambda_{Fv}}{\eta_{vD}z + \lambda_{Fv}} + \eta_{FD} \tag{B3}$$

The solution of Eq. (B2) is

$$\begin{cases} \bar{p}_{1\mathrm{D}} = r_{\mathrm{D}}^{\alpha}\left[AI_n\left(\beta r_{\mathrm{D}}^{\gamma}\right)+BK_n\left(\beta r_{\mathrm{D}}^{\gamma}\right)\right] \\ \bar{p}_{\mathrm{FD}} = CI_0\left(\sqrt{g(z)z}r_{\mathrm{D}}\right)+DK_0\left(\sqrt{g(z)z}r_{\mathrm{D}}\right) \end{cases} \tag{B4}$$

where

$$\alpha=\frac{2-d_{\mathrm{f}}+\theta}{2};\quad \gamma=\frac{\theta+2}{2};\quad n=\frac{2-d_{\mathrm{f}}+\theta}{\theta+2};\quad \beta=\sqrt{\frac{\eta_{1\mathrm{D}}z}{M_1}}\frac{1}{\gamma} \tag{B5}$$

For constant pressure outer boundary, the coefficients *A*-*D* in Eq. (B4) can be determined by the following equation

$$\begin{bmatrix} a_{11} & a_{12} & a_{13} & a_{14} \\ a_{21} & a_{22} & a_{23} & a_{24} \\ a_{31} & a_{32} & a_{33} & a_{34} \\ a_{41} & a_{42} & a_{43} & a_{44} \end{bmatrix}\begin{bmatrix} A \\ B \\ C \\ D \end{bmatrix}=\begin{bmatrix} -1/\left(zM_1\right) \\ 0 \\ 0 \\ 0 \end{bmatrix} \tag{B6}$$

where

$$\begin{cases} a_{11}=\alpha I_n(\beta)+\beta\gamma I_{n-1}(\beta)-n\gamma I_n(\beta);\quad a_{12}=\alpha K_n(\beta)-\beta\gamma K_{n-1}(\beta)-n\gamma K_n(\beta);\quad a_{13}=a_{14}=0; \\ a_{21}=r_{\mathrm{cD}}^{\alpha}I_n\left(\beta r_{\mathrm{cD}}^{\gamma}\right);\quad a_{22}=r_{\mathrm{cD}}^{\alpha}K_n\left(\beta r_{\mathrm{cD}}^{\gamma}\right);\quad a_{23}=-I_0\left(\sqrt{g(z)z}r_{\mathrm{cD}}\right);\quad a_{24}=-K_0\left(\sqrt{g(z)z}r_{\mathrm{cD}}\right); \\ a_{31}=\alpha M_1 r_{\mathrm{cD}}^{\alpha+d_{\mathrm{f}}-\theta-3}I_n\left(\beta r_{\mathrm{cD}}^{\gamma}\right)+\beta\gamma M_1 r_{\mathrm{cD}}^{\alpha+\gamma+d_{\mathrm{f}}-\theta-3}\left[I_{n-1}\left(\beta r_{\mathrm{cD}}^{\gamma}\right)-\frac{n}{\beta r_{\mathrm{cD}}^{\gamma}}I_n\left(\beta r_{\mathrm{cD}}^{\gamma}\right)\right]; \\ a_{32}=\alpha M_1 r_{\mathrm{cD}}^{\alpha+d_{\mathrm{f}}-\theta-3}K_n\left(\beta r_{\mathrm{cD}}^{\gamma}\right)-\beta\gamma M_1 r_{\mathrm{cD}}^{\alpha+\gamma+d_{\mathrm{f}}-\theta-3}\left[K_{n-1}\left(\beta r_{\mathrm{cD}}^{\gamma}\right)+\frac{n}{\beta r_{\mathrm{cD}}^{\gamma}}K_n\left(\beta r_{\mathrm{cD}}^{\gamma}\right)\right]; \\ a_{33}=-\sqrt{g(z)z}I_1\left(\sqrt{g(z)z}r_{\mathrm{cD}}\right);\quad a_{34}=\sqrt{g(z)z}K_1\left(\sqrt{g(z)z}r_{\mathrm{cD}}\right); \\ a_{41}=a_{42}=0;\quad a_{43}=I_0\left(\sqrt{g(z)z}r_{\mathrm{eD}}\right);\quad a_{44}=K_0\left(\sqrt{g(z)z}r_{\mathrm{eD}}\right) \end{cases}$$

(B7)

For closed outer boundary or infinite outer boundary, the coefficients *A*-*D* in Eq. (B4) can be also determined by Eq. (B6) in which a_{43} and a_{44} are modified as

$$a_{43}=\sqrt{g(z)z}I_1\left(\sqrt{g(z)z}r_{\mathrm{eD}}\right);\quad a_{44}=-\sqrt{g(z)z}K_1\left(\sqrt{g(z)z}r_{\mathrm{eD}}\right) \tag{B8}$$

$$a_{43} = I_0\left(\sqrt{g(z)z}r_{\mathrm{eD}}\right); \quad a_{44} = 0 \tag{B9}$$

After solving the Eq. (B4) above, the wellbore pressure in Laplace space is

$$\bar{p}_{1\mathrm{D}}\big|_{r_{\mathrm{D}}=1} = AI_n(\beta) + BK_n(\beta) \tag{B10}$$

Based on the Duhamel's principle, the wellbore pressure considering the wellbore storage and the skin effects is

$$\bar{p}_{\mathrm{wD}} = \frac{z\left[AI_n(\beta) + BK_n(\beta)\right] + S}{z\left[1 + C_{\mathrm{D}}z\left(z\left[AI_n(\beta) + BK_n(\beta)\right] + S\right)\right]} \tag{B11}$$

where S is the skin factor, C_{D} is the dimensionless wellbore storage coefficient.
